# Exploring the Conformational Space of Bcl-2 Protein Variants: Dynamic Contributions of the Flexible Loop Domain and Transmembrane Region

**DOI:** 10.3390/molecules24213896

**Published:** 2019-10-29

**Authors:** Luis A. Caro-Gómez, Jorge L. Rosas-Trigueros, Edgar Mixcoha, José L. Vique-Sánchez, Humberto Gasperin-Sánchez, Claudia G. Benítez-Cardoza, Absalom Zamorano-Carrillo

**Affiliations:** 1 Laboratorio de Bioquímica y Biofísica Computacional, ENMH, Instituto Politécnico Nacional, Ciudad de México 07320, Mexico; lcarog1200@alumno.ipn.mx (L.A.C.-G.); jlv64@hotmail.com (J.L.V.-S.); humberto.gasperin.s@gmail.com (H.G.-S.); beni1972uk@gmail.com (C.G.B.-C.); 2 Laboratorio Transdisciplinario de Investigación en Sistemas Evolutivos, SEPI de la ESCOM del Instituto Politécnico Nacional, Ciudad de México 07738, Mexico; jlrosas@ipn.mx; 3 CONACYT-Instituto Nacional de Psiquiatría, Ramón de la Fuente Muñiz, Ciudad de México 14370, Mexico; edgarmixcoha@gmail.com

**Keywords:** Bcl-2, Bcl-2A1, apoptosis regulation, molecular dynamics simulation, flexible loop domain, intrinsically disordered region, transmembrane domain

## Abstract

Members of the Bcl-2 protein family regulate apoptosis through interactions with several proteins. A critical intrinsically disordered region (IDR) present in some members of the Bcl-2 family is essential for their function. Also, the structural and conformational plasticity of disordered regions is essential for the regulation of the Bcl-2 protein’s activity. Further, some proteins of the family contain transmembrane-helical regions, which anchor them into organelle membranes. Bcl-2, the archetypical member of the family, is characterized by an IDR labeled as a flexible loop domain (FLD) and a transmembrane domain (TMD). Another member of this family is the Bcl-2A1 protein, containing a TMD but lacking the FLD. To our knowledge, this is the first report which characterizes the individual and simultaneous dynamical contributions of FLD and TMD in Bcl-2 and Bcl-2A1 using molecular dynamics simulations (MDS). We examined the conformational spaces of Bcl-2, Bcl-2A1, and two artificial constructs lacking the TMD (Bcl-2ΔTM and Bcl-2A1ΔTM). As the results show, FLD and TMD stabilized each protein independently when they are present. When they coincided, such as in Bcl-2, an additive stabilizing effect is observed. This information is crucial for understanding the structural mechanisms of interaction in the Bcl-2 family.

## 1. Introduction

All multicellular organisms must maintain precise regulation of their cell mass turnover during the entirety of their lifespan to avoid a variety of health disorders such as cancer, autoimmunity, and neurodegenerative dysfunction. Damaged or unwanted cells are eliminated by one of the several types of programmed cell death, with apoptosis being the most common process in mammals [1,2,3]. This mechanism is triggered by the activation of death receptors on the cell surface (extrinsic pathway) or in response to cellular stress involving the mitochondria (intrinsic pathway). The mutual interactions among Bcl-2 family protein members regulate these events [4,5,6]. This family is composed of proteins that either impede (anti-apoptotic) or promote (pro-apoptotic) apoptosis, or regulate pro- and anti-apoptotic members of the family [7,8,9].

Bcl-2, the archetypical member of the family, acts as the primary gatekeeper of the external mitochondrial membrane, supervising the release of cytochrome c from this organelle to the cytosol during apoptosis [10,11,12]. The Bcl-2 gene can potentially encode two proteins: Bcl-2α and Bcl-2β (239 and 205 amino-acid residues in length, respectively) [13,14,15]. Bcl-2α contains a transmembrane domain, which is absent in Bcl-2β. The NMR structure of Bcl-2α (PDB 1G5M) consists of eight α-helices. Two of these (α5 and α6) form the hydrophobic core of the protein surrounded by four amphipathic helices (α1, α2, α3, and α4). Commonly, Bcl-2 (similarly to the rest of the Bcl-2 family members) is described by its BH domains. Bcl-2 contains four BH domains; BH1, BH2, BH3, and BH4 (residues 139–157, 190–206, 100–109, and 14–29, respectively). BH1, BH2, and BH3 form a hydrophobic pocket, which binds BH3 domains of other family members, thereby establishing interactions. BH4 does not participate in protein dimerization, but it may be necessary for anti-apoptotic activity. Bcl-2 also contains an intrinsically disordered region (IDR) located between α1 and α2 and which is composed of 58 amino-acid residues (Gly33-Ala91), which we denote here as a flexible loop domain (FLD). Traditionally, IDRs were considered to be passive segments in protein sequences that “linked” structured domains, but now, we know that these regions are highly flexible to facilitate conformational adaptability, and in consequence, to bind different types of ligands. Furthermore, disordered regions are highly prone to posttranslational modifications (PTMs) that increase their functional states [16,17]. Particularly, FLDs may play major roles in regulating apoptosis through interactions with JNK-1, PKC, PP2A phosphatase, MAP kinase, ubiquitin, PS1, and FKBP38. Some residues within the FLD, such as Asp34, Thr56, Thr69, Ser70, Thr74, and Ser87, are susceptible to phosphorylation. Further, several Caspase-3 cleavage sites are present within the FLD. Phosphorylation and/or caspase-cleavage are important in the activity regulation of Bcl-2 [18,19]. The FLD structure has not been resolved. Nevertheless, due to its importance in the function of Bcl-2, it has been analyzed by molecular dynamics simulations (MDS) in order to describe its flexibility and stability [18]. Additionally, the ninth α-helix at the C-terminus (Leu217-Lys239) of Bcl-2α constitutes the transmembrane domain (TMD). It has been established that the Bcl-2 proteins require the TMD to retain their full anti-apoptotic activity. As a consequence, Bcl-2β proteins lacking the TMD reside in the cytoplasm [13]. There is insufficient information regarding the contributions of the TMD region to the overall conformational dynamics and membrane targeting ability of Bcl-2, as most in silico and in vitro studies have been performed with constructs lacking the TMD [19,20].

Another anti-apoptotic member of the Bcl-2 family is Bcl-2A1, also known as A1/Bfl-1, which is an early-response tissue-specific gene, expressed in several hematopoietic cell lineages, including T-helper lymphocytes, macrophages, and neutrophils [21,22]. Bcl-2A1 is shorter than Bcl-2 (175 amino-acid residues), its structure consists of eight α-helices and contains all four BH domains, the hydrophobic groove, and the C-terminus (also forming a ninth helix which anchors to mitochondria; even though this binding is not mandatory for the anti-apoptotic activity of Bcl-2A1) [23,24,25]. Unlike Bcl-2, Bcl-2A1 lacks the FLD. In vitro studies indicate that the μ-calpain-mediated cleavage of Bcl-2A1 occurs between its α1 and α2, switching the function of this protein from prosurvival to prodeath activities [26]. 

**Figure 1:**
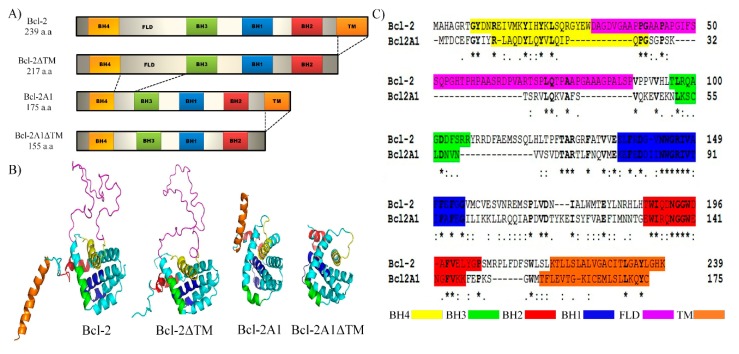
**Sequence and structure of Bcl-2 constructs.** (**A**) Domain architecture of Bcl-2, Bcl-2ΔTM, Bcl-2A1, and Bcl-2A1ΔTM constructs used in this study. (**B**) Three-dimensional structure of Bcl-2 (Bcl-2, Bcl-2∆TM, Bcl-2A1, and Bcl-2A1∆TM). (**C**) Clustal sequence alignment of Bcl-2 and Bcl-2-A1. In all cases, domains are colored as indicated.

Bcl-2 and Bcl-2A1 are fundamental regulators of programmed cell death, and as a consequence, are associated with numerous types of cancer when apoptosis is dysregulated. Furthermore, members of this family represent an important new class of anti-cancer drug therapeutics. Hence, it is imperative to fully understand their dynamic behavior. We are interested in investigating the contributions of TMD and FLD domains (individually and collectively) to the stability, conformational dynamics, and function of Bcl-2 family proteins. Although it is known that proteins belonging to the Bcl-2 family (Bcl-w, Bcl-xl, and Bcl-2) are biologically active when anchored to the mitochondrial outer membrane, structural details of the dynamic behavior of their domains before interacting with pro or anti-apoptotic proteins or prior to being inserted into the membrane are still missing. The study of these domains is difficult because of the lack of coordinates. To the best of our knowledge, here, we have described, for the first time, the combined participation of FLD and TMD regions by exploring the conformational space of Bcl-2, Bcl-2A1, and two artificial constructs lacking the C-terminal domain (Bcl-2ΔTM and Bcl-2A1ΔTM) (Figure 1A,B). This information contributes to our understanding of the regulation of apoptosis by these structural elements.

## 2. Results

### 2.1. Sequence and Structure Analysis

Sequence alignment of Bcl-2 and Bcl-2A1 (Figure 1C) revealed 22.17% identity and 37.23% similarity between them (percentages calculated from full-length sequences). The BH domains were the most conserved regions; BH1 and BH2 domains exhibited 35.6% and 55% identity, respectively. BH4 domain showed 33.3% identity between Bcl-2 and Bcl-2A1. With respect to the BH3 domain, only two residues, Leu97 and Asp103 (residue numbers according to Bcl-2 sequence), were conserved between Bcl-2 and Bcl-2A1. In several pro-apoptotic and anti-apoptotic proteins, the same residues are also conserved [27]. In contrast, the C-terminal region is highly variable between these proteins, as their identity is 12.5%.

The structures of human Bcl-2α and Bcl-2A1 have been reported, but the constructs do not include certain residues [18]. The PDB file 1G5M, corresponding to Bcl-2α structure, lacks residues 51–91, which are present within the FLD, and residues 208–239, which constitute the TMD region. In Bcl-2A1, the missing residues are 14–19, which are present within the loop connecting the BH4 and BH3 domains, and 26 residues found at the C-terminus (150–175). The coordinates of the proteins, including missing residues in the reported structures, were obtained using the I-TASSER server.

Constructs corresponding to Bcl-2∆TM and Bcl-2A1∆TM were obtained by deleting the residues present in the TMDs from the corresponding sequence and then submitting the resulting sequence to the I-TASSER server. For all constructs, we observed the archetypical scaffold of the Bcl-2 family, composed of two hydrophobic helices (α5 and α6), flanked by six amphipathic helices (α1, α2, α3, α4, α7, and α8). As expected, the FLD region of Bcl-2 did not exhibit regular secondary structure and protruded away from the central core. In the corresponding position, Bcl-2A1 had a short mobile loop 17 residues in length and lacking regular secondary structure. The C-terminal regions of Bcl-2 and Bcl-2A1 were modeled as α-helices composed of 22 or 20 amino-acid residues, respectively. In both proteins, the C-terminal regions extended away from the central core of the protein. All the possible pairs of proteins were compared using RMSD values. Two types of methods to carry out the quantitative analysis of the contribution of the FLD and TM domains by RMSD calculation can be used. (I) Sequence-dependent methods of protein structure comparison assume a strict one-to-one correspondence between target and model residues; and (II) sequence-independent methods whose structural superimposition is performed independently, followed by the evaluation of residue correspondence obtained from such superimposition [28,29]. We have used a sequence-dependent method to obtain two values of RMSD in each comparison, using the Align method implemented in Pymol software [30]. Align performs a sequence alignment followed by a structural superposition, and then carries out none or a defined number of cycles of refinement to reject structural outliers found during the fit. Thus, two RMSD values were obtained with or without refinement, respectively. The second value included five cycles of refinement and was labeled as RMSDr. Considering the RMSD, the absence of ΔTM produces the largest variation in Bcl-2 (6.979 Å, RMSD), and considering the RMSDr, the lack of FLD is the most influencing factor (1.721 Å). Interestingly, the effect on the RMSDr due to the absence of FLD is similar to those obtained by the absence of two both domains, using the refinement cycles (1.721 Å ≈ 1.737Å). It is to note that the alignment takes into consideration as many atoms as possible for a given pair of structures; therefore, the comparison between these values might not be fully accurate due to differences in sequence (mainly sequence length). Nevertheless, it is remarkable that the overall scaffold is almost fully superimposable. Furthermore, FLD of Bcl-2 and the short loop of Bcl-2A1 lie on the same location (Figure S1).

### 2.2. Molecular Dynamics Simulation (MDS)

**Figure 2:**
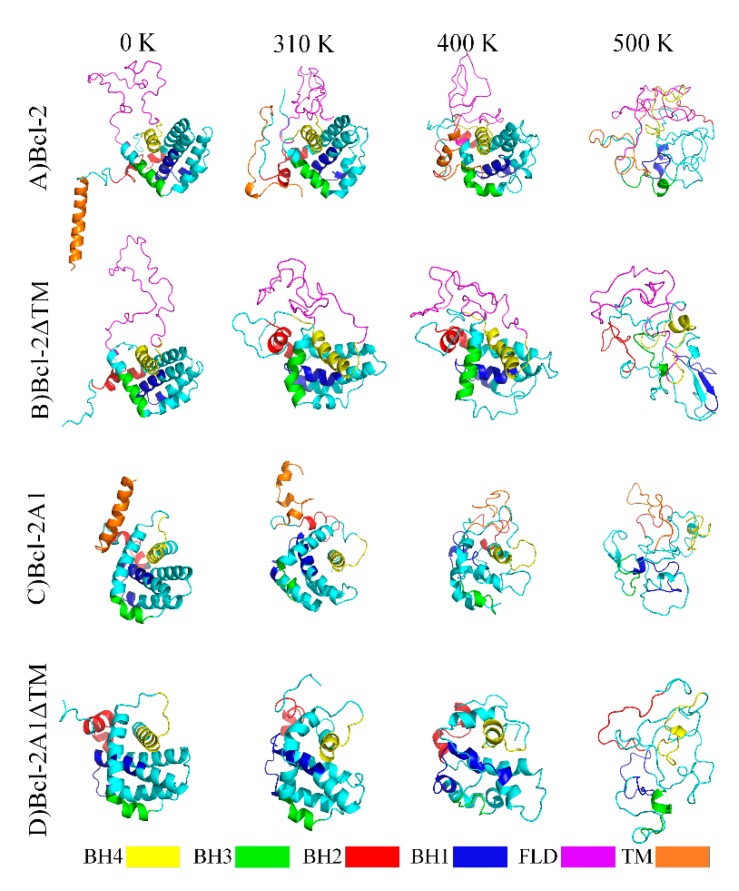
The middle structures obtained from the main clusters of MDS at 310, 400, and 500 K for (**A**) Bcl-2, (**B**) Bcl-2ΔTM, (**C**) Bcl-2A1, and (**D**) Bcl-2A1ΔTM. Domains are colored as indicated.

The MDS trajectories were analyzed with tools included in GROMACS. Several conformational parameters were analyzed, such as root mean squared deviation (RMSD), root mean squared fluctuation (RMSF), radius of gyration (RG), solvent accessible surface area (SASA), and hydrogen bonds (Hb); the interactions between residues were described by a contact map, and the time-course evolution of the secondary structure were also described. In all cases, substantial conformational changes occurred in the first ns of simulation before reaching a plateau. The abrupt changes are considered to correspond to equilibration time. Therefore, the description presented here corresponds to the behaviors of proteins when they have reached equilibrium, i.e., production time. Clustering analysis allowed us to find the largest cluster for each trajectory [31,32]. The main characteristics of the set of clusters and the largest ensemble in each simulation are described in Supplementary Table S1. It is observed that when FLD and TM domains are absent, equipartition is increased in the proportion of the clusters formed by each protein. Thus, the preponderance of the main cluster is lost. The central structures of these clusters are shown in Figure 2. 

### 2.3. Bcl-2 Protein

**Figure 3:**
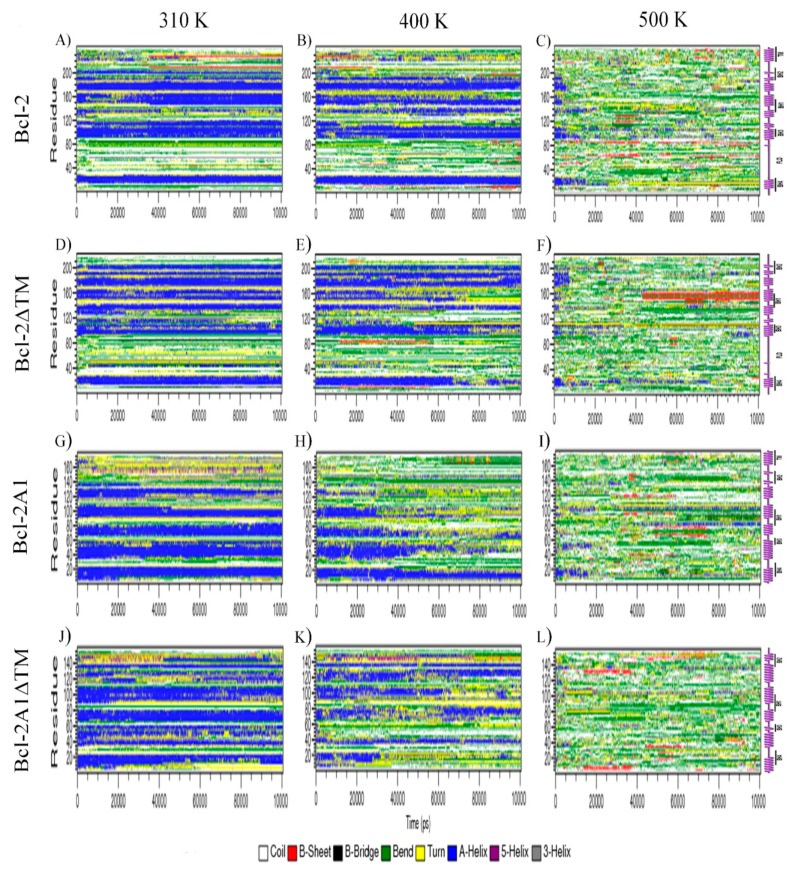
Time-course evolution of the secondary structure of Bcl-2 (**A**–**C**), Bcl-2ΔTM (**D**–**F**), Bcl-2A1 (**G**–**I**) and Bcl-2A1ΔTM (**J**–**L**). A representation of the secondary structure with labels for the main domains is included on the right. The color code of each secondary structure element, according to the Dictionary of Protein Secondary Structure (DSSP) classification, is shown at the bottom of the image.

**Figure 4:**
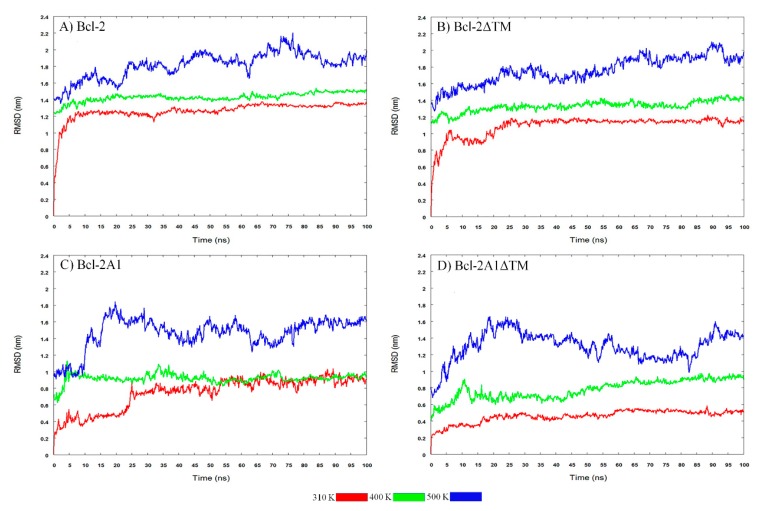
Time evolution of C-α root mean square deviations (RMSD) of (**A**) Bcl-2; (**B**) Bcl-2ΔTM; (**C**) Bcl-2A1, and (**D**) Bcl-2A1ΔTM. The color indicates the temperature of each molecular dynamic (MD) simulation, as stated.

**Figure 5:**
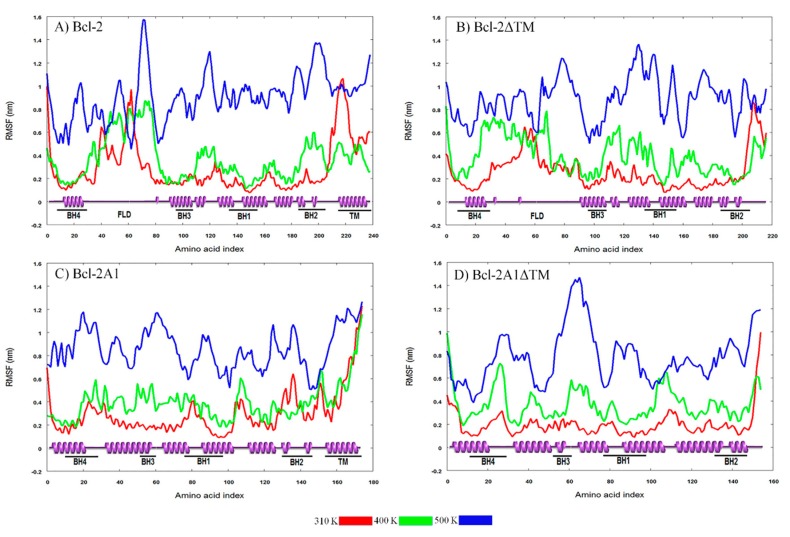
Root mean square fluctuations (RMSF) of the C-α atoms of (**A**) Bcl-2; (**B**) Bcl-2ΔTM; (**C**) Bcl-2A1, and (**D**) Bcl-2A1ΔTM, as a function of residue number. At the bottom of each plot, a representation of the secondary structure with labels for the main domains is included. Colors indicate the temperature of the MD simulation.

Conformational and topological parameters of Bcl-2 protein at different temperatures are shown in Figure 2A, panels A of Figures 4 and 5, and Supplementary Figures S6–S9. In addition, representative images obtained every 20 ns of the simulation are shown in Supplementary Figure S2. It is observed that most of the secondary structure of Bcl-2, formed by eight α-helices, was maintained without any substantial change during the course of the MDS (Figure 3A). Meanwhile, the ninth α-helix at the C-terminus was destabilized from the very first ns of MDS; afterward, this helix lost helicity and oscillated mainly between unstructured and helical structures up to the end of the simulation. In addition, some regions within the TMD acquired beta-sheet structures after 40 ns. Remarkably, the FLD folded on itself and became closer to the central core.

These protein motions are associated with RMSD values (Figure 4A), which change importantly during the first ten ns of MDS, and after this time, reached the characteristic equilibrium plateau (around 1.2 nm). The regions with the highest mobilities within the protein were TMD and FLD (Figure 5A), reaching maximum RMSF values of 0.90 and 1.05 nm, respectively. In addition, RG and SASA values decayed from 2 to 1.8 nm and from 145 to 130 nm^2^, respectively (Supplementary Figures S6A and S7A). Concomitantly, the number of Hb increased (Supplementary Figure S8A). 

When we applied higher kinetic energy by increasing the temperature conditions of the MDS, Bcl-2 protein displayed significant conformational changes. At 400 K, Bcl-2 also preserved the helical content in its BH domains. The main changes observed during the first 35–40 ns of simulation belonged to the FLD and TMD regions, which adopted conformations that are more compact, and which approach the protein core, while covering BH1 and BH2 (mainly) and part of the BH3 domains. The FLD domain did not remain attached to the core of Bcl-2 during the whole period of MDS. On the contrary, the TMD region approached the main core and maintained this proximity during the whole duration of the MDS. These movements caused a reduction in RG and SASA values. The average RG and SASA values, in this case, were 1.78 nm and 121 nm^2^, respectively. Concomitantly, the number of Hb was augmented. When the temperature was increased from 310 to 400 K, on average, 9 Hb were gained. This was also observed by comparing contact maps obtained at 310 and 400 K. A higher number of interactions can be observed at 400 K than at 310 K. Generally speaking, these changes brought the main domains (BH1, BH2, and BH3) closer together. Furthermore, we observed how the TMD region interacts with BH3, BH4, and BH1, and how the FLD approaches BH2 (Supplementary Figures S9A and S9B).

MDS at extreme temperatures provided information concerning the stability and the unfolding pathway in the context of atomic motions [33]. At 500 K, Bcl-2 showed significant loss of native conformation in most of the structural domains and important fluctuations in RMSD, RG, SASA, and Hb values. BH3 and BH4 kept their native secondary structure, at least during the first 5 ns of simulation. SASA and RG values increased due to the extended conformations, which can be related to a reduction in Hb.

### 2.4. Bcl-2ΔTM Protein

Bcl-2ΔTM was one of the artificial constructs in which the coordinates of the TMD region were deleted. The results of MDS for this construct, at different temperatures, are shown in Figure 2B and panels B of Figure 4 and Figure 5, and Supplementary Figures S6–S9. At 310 K, the Bcl-2ΔTM construct showed large conformational fluctuations during the initial ns of simulation until reaching a stable conformation, similar to the behavior observed for Bcl-2. Snapshots obtained every 20 ns of MDS are shown in Supplementary Figure S3. In the snapshots, and in the secondary structure of Bcl-2ΔTM, we can observe how the conformation of the eight α-helices is maintained, without apparent changes during the 100 ns of simulation, except for the helix formed by residues 125–132, which tends to lose helicity (Figure 3D). The evolution of RMSD values shows two stages, the first one observed before 25 ns of simulation (0.98 nm), and the second one, which is maintained up to the end of the simulation (1.18 nm) (Figure 4B). The main change observed between the two stages is a compaction of the protein, mainly of the FLD, which shows the most extensive motions within the protein, reaching maximum RMSF values of around 0.80 nm, similar to those achieved by the Bcl-2 protein (Figure 5B). This compaction is also detected by the reduction of RG (from 1.87 nm to 1.81 nm) and SASA (128.5 from to 120 nm^2^) values (Supplementary Figures S6B and S7B), and a small increment of approximately 5 Hb (Supplementary Figure S8B). 

At 400 K, the most prominent change occurs within the FLD region, which is compacted and approaches the central protein core, hiding some BH domains from the solvent. This behavior is similar to that observed for Bcl-2; these conformational changes cause a reduction in RG and SASA values, in comparison to the values obtained at 310 K. On average, the RG and SASA values were 1.78 nm and 115 nm^2^, respectively. The compaction of Bcl-2ΔTM is also accompanied by the formation of approximately 5 new Hb. On comparing the MD simulations obtained at 310 K and 400 K, we observed an increment in the interactions between the BH domains of the protein, especially between the BH2 domain and the FLD region (Supplementary Figure S9D,E). 

At 500 K, Bcl-2ΔTM showed a significant loss of secondary structure in most of the structural domains (BH domains) after 8 ns at this temperature. Interestingly, we observed that residues 150–163 acquired a β-strand conformation during the second half of the simulation time. This unfolding behavior was confirmed by the reduction of Hb and increments of RG and SASA.

### 2.5. Bcl-2A1 Protein

Analyses of the trajectories of the simulations for Bcl-2A1 are shown in Figure 2C and panels C of Figure 4 and Figure 5 and Supplementary Figures S6–S9. Some representative snapshots obtained every 20 ns of the simulation are shown in Supplementary Figure S4. Bcl-2A1 at 310 K retains most of its secondary structure during the 100 ns of simulation (Figure 3G), except for helices formed by residues 155 and 175, which are reduced in their helical content. This behavior of the TMD region of Bcl-2A1 is identical to that observed for the TMD region of Bcl-2. The plot of RMSD shows a plateau after 50 ns, but before reaching this stable conformation, there are two intermediate states from 0 to 25 and from 25 to 50 ns (Figure 4C). The maximum values observed (1.2 nm) in the RMSF plot corresponded to the TMD region (Figure 5C). The average values of RG, SASA, and Hb were 1.72 nm, 107.8 nm^2^, and 117, respectively (Supplementary Figures S6C, S7C and S8C). 

Bcl-2A1 exhibited critical conformational changes at 400 K. The most relevant was the approach of the TMD to the main core of the protein, as well as the loss of secondary structure in some α-helices present between residues 40–60, 65–80, 90–105, and 115–140, some of them in the firsts ns of MDS and others after 50 ns, accompanied by the expansion of the hydrophobic core. In this case, the RMSD did not show significant changes. SASA and RG values seemed to be constant during the first 50 ns of simulation, but it is important to point out that these geometrical parameters represent changes in the overall conformation. In this case, the snapshots show the compaction of one region of the protein (TMD), concomitantly with the expansion of the main core. During the second half of MD simulation, TMD seems to be even more compact, while the hydrophobic core expands further. This conformational change implies a reduction of RG and SASA passing from 1.69 to 1.62 nm and from 104 to 98 nm^2^, respectively. The compaction of the TMD is associated with an increment of approximately 5 Hb. The contact map of Bcl-2A1 revealed that the TMD region interacts with its close neighbors and the BH2 domain (Supplementary Figure S9H). At 500 K, most of the BH domains lose their secondary structure, except for BH3 and BH4 domains, which maintain part of their native secondary conformation during the first 15 ns of simulation.

### 2.6. Bcl-2A1ΔTM Protein

Bcl-2A1ΔTM lacks both FLD and TMD regions. Figure 2D and panels D of Figure 4 and Figure 5, and Supplementary Figures S5–S9 show the results obtained for this protein. At 310 K, Bcl-2A1ΔTM maintains most of its native-like secondary structure throughout the simulation period, the conformation of some regions between residues 5–15, 40–50, 90–95, 115–125, and 145–155 was observed to fluctuate between α-helices and random coils (turns and bends, Figure 3J). Bcl-2A1ΔTM showed a gradual increment in RMSD values from 0.24 to 0.5 nm, until reaching equilibrium at around 60 ns (Figure 4D). In this case, this protein construct showed the longest time to reach a plateau at 310 K. RMSF values are on average around 0.2 nm for all BH domains. In this case, the highest values of RMSF were observed for the loops that connect the α-helices (Figure 5D). RG and SASA did not show appreciable changes; they oscillated around 1.55 nm and 93.5 nm^2^, respectively (Supplementary Figures S6D and S7D).

The RG and SASA values for Bcl-2A1ΔTM, at 400 K, did not show considerable change; they decreased slightly (on average, around 0.01 nm and 1.2 nm^2^, respectively). The number of Hb was increased from 100 to 105 (Supplementary Figure S8D), and Bcl-2A1ΔTM showed a reduced number of contacts involving BH3 and BH4 domains. However, an increment of contacts was detected between BH2 and BH4 domains (Supplementary Figure S9K).

The Bcl-2A1ΔTM construct appeared to be the least stable protein of all, since at high temperatures it entirely loses secondary structure, even during the first ns of the simulation at 500 K. Since Bcl-2A1ΔTM contains neither TMD nor FLD regions, it does not show the compaction of these domains as observed for the other constructs. Furthermore, taking into account the time to reach equilibrium and the maintenance of the secondary structure, this is the least stable protein, because its secondary structure is partially lost even at 400 K, and the equilibrium of RMSD values was achieved after 60 ns at 310 K.

Therefore, we propose that the FLD and TMD domains stabilize the Bcl-2 protein and that this stabilizing effect is additive.

### 2.7. Essential Dynamics

In previous sections, we observed that both the FLD and TMD regions approach the central core of the protein, in those proteins that have at least one such domain. The conformational changes were studied identifying global collective motions, which are most relevant to the activity of the protein by using essential dynamics [34]. 

**Figure 6:**
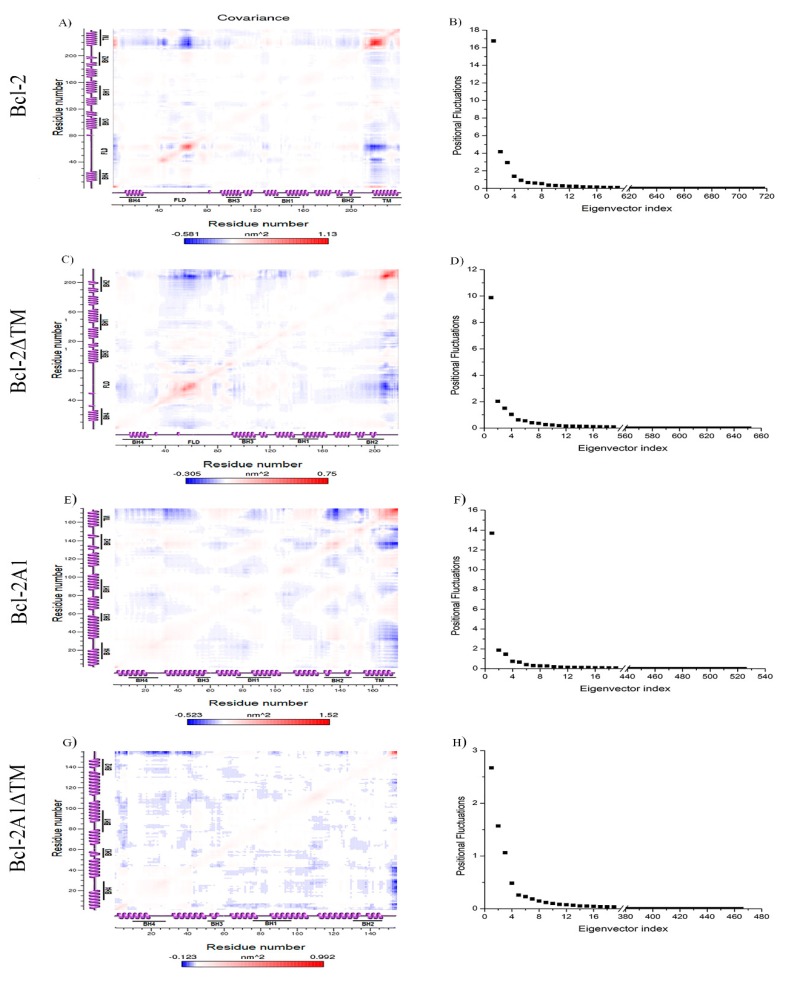
The covariance matrices (**A**,**C**,**E**,**G**) shows correlated (red) and anti-correlated (blue) motions between atoms. The color intensity indicates the amplitude of the RMS fluctuations (see the color bar). The corresponding eigenvalues fluctuations are shown in plots (**B**,**D**,**F**,**H**) in decreasing order of magnitude as obtained from the respective C-α coordinate covariance matrix.

The covariance matrix shows the degree of collinearity in atomic motions or inter-residue cross-correlations. In Figure 6A,C,E,G, the covariance matrices are shown for each of the constructs studied; these arrays identify correlated or anti-correlated regions at different levels. We observe that FLD residues 40–80 and TMD for Bcl-2 show high cross-correlation with residues in their vicinity. We detected that the amino acid residues belonging to each of these regions show a high degree of collinearity. However, these two regions are anti-correlated. In the case of Bcl-2ΔTM, we detected a moderate correlation within FLD residues 35–75. Moreover, we observed a weak correlation between residues 105–118. Raghav and co-workers studied a construct of Bcl-2, lacking the TMD, quite similar to the construct Bcl-2ΔTM, studied here. They observed a correlation of the FLD and BH3 domain [20]. For Bcl-2A1, we observed a high correlation between the residues within the TMD region, while moderate to weak collinearity was detected in regions involving residues 55–75 and 105–125. In contrast, for this construct, we identified strong anti-correlation between the TMD and the regions composed of residues 20–50, 85–103, and 130–152. For Bcl-2A1ΔTM, we could not encounter correlated movements; in this case, we only observed anti-correlated movements between different regions within the protein. Additionally, in Figure 6B,D,F,H, we observed that more than 90% of the collective motion can be described by the first ten eigenvectors, indicating that the most significant motions detected were mainly based on overall positional fluctuations.

## 3. Discussion

### The Contribution of FLD and TMD to Bcl-2 Family Structure and Function Regulation

All proteins studied here exhibited conserved α-helices during the time span of MDS at 310 K. This observation indicated the existence of networks of strong hydrophobic interactions as well as Hb within all BH domains, which are essential in preserving the 3D structure of Bcl-2 proteins. Maintaining all BH domains, and mainly BH3, is crucial for these proteins because BH domains are responsible for most interactions with other proteins of the Bcl-2 family [9].

Some Bcl-2 family proteins, such as Bcl-2, Bcl-xL, and Bcl-w, display loops of variable length, connecting α1 and α2 [35,36]. In the case of Bcl-2 and Bcl-xL, the loops are quite long (58 and 49 residues long, respectively) and disordered, as judged by the lack of electron density in the X-ray structures and ^1^H-^15^N NOE data. In contrast, Bcl-w displays a shorter loop (13 residues in length) compared with its longer counterparts, and shows a well-defined structure, and is relatively immobile, according to ^15^N-relaxation data [36]. This short loop of Bcl-w resembles, in length, the short loop of Bcl-2A1. 

In this work, the sequences and structures of Bcl-2 and Bcl-2A1 were compared for the first time. We found that these proteins are highly conserved in both sequence and conformation. The main difference observed resides in the length of the loop connecting α1 and α2. In Bcl-2, this region has been well recognized as an IDR. In contrast to the attention Bcl-2 has received, the loop region of Bcl-2A1 has been scarcely studied. 

We were curious to further investigate whether this region could be described as an IDR. According to analysis using IUPred, the loop of Bcl-2A1 showed the highest IDR propensity values within the sequence, although they do not reach the cut-off values to consider this region as an IDR (Supplementary Figure S10B) [37]. Furthermore, the results of MDS performed at 310 and 400 K showed that this loop has the highest RMSF along the sequence. 

Considering these observations, an important question emerges. What would be the biological relevance of the length and flexibility of the loop connecting α1 and α2 in Bcl-2 family proteins? Three possible regulation mechanisms revealed by MDS results can provide insight into this issue. 

The first possible answer could be related to the regulation of the function of Bcl-2 protein. It is interesting to note, that one the most significant structural rearrangements observed in our results obtained at 400 K, in both Bcl-2 and Bcl-2ΔTM, was that FLD folds in on itself, adopting a more compact conformation and moving towards the protein core in the first third of the simulation (Supplementary Figures S2 and S3). Similar results have been reported in previous reports. The conformational changes observed, showing the propensity of FLD to fold on itself and to move towards the core are, not dependent on the force-field or program used for MDS analysis [19,20]. When FLD approaches the central core, Bcl-2 and Bcl-2ΔTM become more compact. Furthermore, FLD blocks access to BH1, BH2, and part of BH3 domains, thus impeding the interaction of these domains with other members of the family. These results suggest that FLD dynamics participate in the regulation of Bcl-2 function by hindering ligand binding. It has been confirmed experimentally, that residue Ser70 of FLD is phosphorylated. This post-translational modification may improve the anti-apoptotic function of Bcl-2. In addition, multisite Bcl-2 phosphorylation induced by anti-mitotic drugs like paclitaxel may inhibit Bcl-2. Furthermore, MD simulation of constructs of Bcl-2 either unphosphorylated of phosphorylated at Ser70 has demonstrated to have different collective movements within the FLD, indicating that the FLD and BH3 might regulate the physiological activity of Bcl-2 [38,39].

A second possible answer could be associated with the half-life reported for Bcl-2 and Bcl-2A1 proteins that are 24 h and 30 min, respectively. It is well known that IDR regions frequently show regulatory elements such as phosphorylation and PEST sequences (sequences enriched in proline [P], glutamic acid [E], serine [S], and threonine [T]) [35]. These are susceptible to protein degradation either through μ-calpain cleavage or ubiquitination-triggered protein degradation, thus governing the half-life of the proteins involved [40]. We analyzed the sequences of Bcl-2 and Bcl-2A1 using the epestfind server to identify and locate PEST sites within these regions [41]. The regions corresponding to residues (^26^RGYEWDAGDVGAAPPGAAPAPGIFSSQPG^55^) in Bcl-2 and (^21^LQIPQPGSGPSK^32^) in Bcl-2A1 were recognized as potential PEST sites. They correspond to FLD and the short loop connecting α1 and α2 in each protein. When FLD folds on itself and approaches the main core, some of these residues become less accessible to proteases. On the contrary, the short loop of Bcl-2A1 did not show conformational changes devoted to hiding the potential PEST sites. We believe that this might explain the differences of the half-life reported for these proteins [21]. (Supplementary Figure S11). The μ-calpain cleavage site is located within the short loop of Bcl-2A1. This cleavage site remains exposed during all MDS, thus allowing N-terminal truncation and producing death-promoting fragments of Bcl-2A1 upon μ-calpain digestion [26,40].

With respect to the contribution of the TMD region to the function of Bcl-2 family proteins by MDS at 400 K, we observed that TMD regions of both Bcl-2 and Bcl-2A1 moved towards the main core and interacted mainly with BH3 in Bcl-2 and BH2 in Bcl-2A1 (Supplementary Figures S2 and S4). Some reports indicate that the TMD region can block access to the binding sites (BH3) of either pro- or anti-apoptotic proteins such as Bcl-2, Bcl-w, Bcl-xL, and Bax. For example, kinetic data suggest that the TMD of Bcl-w and Bcl-xL modulate pro-survival activity by regulating ligand access to the binding groove corroborating the behavior of the TMD observed in MD simulations [42,43,44].

It is interesting to note that the Bcl-2A1ΔTM artificial construct showed a different behavior than that observed for the other proteins in this study. It was the only construct that showed an increment in the mobility of all regions simultaneously while increasing the temperature from 310 K to 400 K. It showed a slight increase in the value of RG and SASA. This behavior could be due to the lack of FLD and TM domains that contribute to interactions that otherwise stabilize and reduce the expansion of the molecule. In addition, we observed a substantial structural change occurring in the BH3 domain, which entirely loses its helicity from the start of the MDS at 400 K. 

In summary, both TMD and FLD showed a tendency to approach the core when they are present in the protein. Bcl-2 has both domains, located on opposite sides of the structure, and their movements are highly anti-correlated. However, there were not any new contacts between TMD and FLD. Movement of both regions towards the main core involves a more significant reduction of RG and SASA compared to the single domain approximation towards the core. In addition, there is a larger increment of Hb, as well as other non-covalent interactions. Therefore, we conclude that FLD and TM together stabilize proteins of the Bcl-2 family. Inclusion of the lipid environment to the C-termini of Bcl-2 and Bcl-2A1 would be an important aspect to consider when studying the impact of the loop on BH domain dynamics, which will be addressed in future work. 

## 4. Materials and Methods

To obtain the initial coordinates for MDS at 310 K, we constructed three-dimensional models of Bcl-2, Bcl-2∆TM, Bcl-2A1, and Bcl-2A1∆TM, using the I-TASSER server [45]. For Bcl-2, the full-length sequence of human Bcl-2 from the National Center for Biotechnology Information (NCBI) (http://www.ncbi.nlm.nih.gov) with accession number NP_000624.2 was used as the input sequence. The three-dimensional structure of human Bcl-2 that is reported in the Protein Data Bank (PDB, http://www.rcsb.org/pdb/home/home) with the PDB-ID 1G5M was employed as a template for homology modeling. Missing residues (FLD (34V-P91) and TMD (208P-K239)) were modeled by a combination of ab initio procedures and protein threading. For Bcl-2A1 and Bcl-2A1∆TM, sequences with the accession numbers CAG46735.1 from NCBI and PDB-ID 2VM6 from PDB were used. The full-length coordinates of Bcl-2A1 also had to be modeled by I-TASSER because there were missing residues in the 2VM6 template, namely, (25Q-P30) and (150P-C175). For Bcl-2∆TM and Bcl-2A1∆TM, the corresponding C-terminal regions were eliminated from each of the original sequences; these included (217K-K239) and (155T-C175). Afterward, the abbreviated sequences were submitted to the I-TASSER server. Analysis of the quality of the structures included Ramachandran plot generation and analysis of the parameters obtained by PROCHECK and ProQ servers to achieve the best models [46,47]. The results obtained by PROCHECK and ProQ servers provided confidence (Table 1) that the three-dimensional models presented high structural quality and, therefore, were suitable to perform MDS upon the refinement of the structures at 310 K.

All MDS were performed with GROMACS 4.5.7 [48,49] using the OPLS-AA force field [50]. We used the all-bond constraints LINCS algorithm and the leap-frog algorithm for integrating Newton’s equations [51]. Each protein was solvated in a rectangular box of Single Point Charge (SPC) water [52]. A minimum distance of 1 nm from each protein to the edge of the box was established, and periodic boundary conditions were also applied. To neutralize the system, counter-ions were added, and water molecules were removed if they overlapped with the ions. All system data are shown in Table 2. During energy minimization, the steepest descent algorithm was used until convergence. Further equilibration of the system was accomplished in 5000 steps (10 ps) of MDS with restricted protein atoms and NVT conditions. MDS were performed with a time step of 2 fs under NPT conditions such that the size of the box fluctuated to maintain the pressure at a constant value. Every 250 steps, the coordinates for the whole system were saved, and after every ten steps, the neighbor lists were updated. The PME algorithm was used for electrostatic interactions with a cut-off of 1 nm [53]. The van der Waals interactions used 1 nm as a single cut-off. Temperature and pressure coupling were performed with the Nosé-Hoover algorithm [54,55] and the Parrinello-Rahman algorithm [56,57], respectively. After stabilization of the system, the potential energy was conserved during MDS in all cases. 

The protocol commenced with 100 ns of MDS at 310 K without any atom fixing. The starting point for MDS at 400 K was the latter trajectory obtained at 310 K. The system was gradually heated during 2 ns. From 310 to 400 K. Afterwards, 100 ns of MDS at 400 K were carried out. Finally, a second gradual heating was performed from 400 to 500 K for 2 ns, before completing the MDS at 500 K. The results were analyzed using tools included in GROMACS. Graphical representations of the protein were obtained using PyMol and VMD programs [30,58].

## 5. Conclusions

The contribution of FLD and TMD regions is cumulative in maintaining the overall structure of Bcl-2 family proteins. In Bcl-2, FLD folds on itself and moves closer to the central core at 400 K during the first 35–40 ns of MDS, whereas afterward, it moves apart from the main core, which remains compact. Meanwhile, the TMD loses helicity and also approaches the nucleus of the protein at the same temperature. When both domains are near the core, they concomitantly approach each other, shielding BH1, BH2, and BH3 domains. These events might promote stabilization and compaction of Bcl-2 through the formation of new interactions. Furthermore, these conformational changes might play important roles in protein regulation and even induce dramatic changes in the function from anti-apoptotic to pro-apoptotic of Bcl-2 family proteins.

## Figures and Tables

**Table 1:** Model performance in terms of structural analysis.

Servers	Bcl-2	Bcl-2∆TM	Bcl-2A1	Bcl-2A1∆TM
Procheck	94.6%	96%	94.2%	98%
ProQ * (LGscore)	4.48	4.34	4.49	3.61

* LGscore > 1.5: fairly good model; LGscore > 2.5: very good model; and LGscore > 4: extremely good model.

**Table 2:** System data for each construct used in molecular dynamics simulations.

Construct	Number of Na^+^1	Total Number of Atoms	Number of Water Molecules	Box Size (L × W × H, nm)
Bcl-2	1	57,757	18,033	7.993 × 5.737× 5.446
Bcl-2∆TM	3	54,380	17,019	6.208 × 5.631 × 7.066
Bcl-2A1	2	26,447	7873	4.519 × 4.799 × 3.948
Bcl-2A1∆TM	2	26,178	7875	4.563 × 4.236 × 4.398

## Supplementary Materials

The following are available online at https://www.mdpi.com/1420-3049/24/21/3896/s1, Figure S1: Diagonally symmetric table showing the superposition of initial structures and two assessments of RMSD and RMSDr. The RMSDr values were calculated, including 5 cycles of refinement to reject structural outliers. Images and RMSD values were obtained by Pymol. The distances between aligned C-alpha atom pairs are stored as B-factors of these residues, which are dyed by a color spectrum, with blue specifying the minimum pairwise RMSD and red indicating the maximum. Unaligned residues are colored gray. Figure S2: Representative snapshots of the conformational changes observed at 310, 400, and 500 K for Bcl-2 followed each 20 ns. Figure S3: Representative snapshots of Bcl-2ΔTM, along MD simulations at 310, 400, and 500 K, followed each 20 ns. Figure S4: Representative snapshots of the conformations adopted by Bcl-2A1 observed each 20 ns of MD simulation at 310, 400, and 500 K. Figure S5: Characteristic snapshots of Bcl-2A1ΔTM observed each 20 ns of MD simulation at 310, 400, and 500 K. Figure S6: Radius of gyration (RG) of structures A) Bcl-2; B) Bcl-2ΔTM; C) Bcl-2A1 and D) Bcl-2A1ΔTM as a function of simulation time. Figure S7: Solvent accessible surface area (SASA) of A) Bcl-2; B) Bcl-2ΔTM; C) Bcl-2A1 and D) Bcl-2A1ΔTM, with respect to simulation time. The color code defined for the different temperatures is shown. Figure S8: Hydrogen bonds (Donor-Acceptor within a cut-off distance of 0.35 nm and angle of 30°) of A) Bcl-2; B) Bcl-2ΔTM; C) Bcl-2A1 and D) Bcl-2A1ΔTM, as a function of simulation time. The color code for each temperature is shown. Figure S9: Contact maps formed between the residues of each of the proteins Bcl-2 (panels A, B and C); Bcl-2ΔTM (panels D, E and F); Bcl-2A1 (panels G, H and I), and Bcl-2A1ΔTM (panels J, K and L). They were calculated from the minimum distance matrix (0–1.5 nm). We show average values observed along the whole simulations at 310, 400, and 500 K. Figure S10: Prediction of protein disorder using the IUPred web server for A) Bcl-2 and B) Bcl-2A1. Figure S11: Porcupine plot of A) Bcl-2 and B) Bcl-2A1, illustrating the motion along the first eigenvectors (EV) obtained from PCA. The regions recognized as potential PEST sites in Bcl-2 and Bcl-2A1 were colored in green. Table S1 Descriptive data of clusters of conformations for each protein, at different temperatures. Ensemble of structures based on the RMSD using the clustering tool of GROMACS and the GROMOS clustering algorithm.
