# Supplementary material for: Exploring the Conformational Space of Bcl-2 Protein Variants: Dynamic Contributions of the Flexible Loop Domain and Transmembrane Region
Source: Molecules. 2019 Oct 29;24(21):3896. doi: 10.3390/molecules24213896 (PMC6865210; doi:10.3390/molecules24213896)
Supplement: Supplementary file 1 [file molecules-24-03896-s001.zip › Suplementaries/Table_S1.docx]

Table S1. Descriptive data of clusters of conformations for each protein, at different temperatures.

| Protein | Temperature  (K) | Number of found clusters | Number of structures for the matrix | Size of main cluster | % of main  cluster | Members of the  cluster (Time ns) | RMSD of main cluster |
| --- | --- | --- | --- | --- | --- | --- | --- |
| Bcl2 | 310 | 4 | 901 | 678 | 69.1 | 32-100 | 0.283 |
|  | 400 | 12 | 1001 | 420 | 41.9 | 38-83 | .358 |
|  | 500 | 13 | 1001 | 238 | 23 | 40-70 | 0.792 |
| Bcl2∆TM | 310 | 11 | 926 | 640 | 69.1 | 27-94 | 0.207 |
|  | 400 | 9 | 1001 | 391 | 39 | 3-46 | 0.353 |
|  | 500 | 15 | 1001 | 265 | 26.4 | 52-82 | 0.814 |
| Bcl2-A1 | 310 | 10 | 951 | 538 | 56.5 | 30-100 | 0.281 |
|  | 400 | 13 | 1001 | 464 | 46.3 | 53-100 | 0.317 |
|  | 500 | 16 | 1001 | 227 | 22.6 | 44-76 | 0.747 |
| Bcl2-A1∆TM | 310 | 7 | 976 | 338 | 34.6 | 16-52 | 0.208 |
|  | 400 | 13 | 1001 | 370 | 36.9 | 50-100 | 0.365 |
|  | 500 | 11 | 1001 | 333 | 32.2 | 47-85 | .0749 |

*Ensemble of structures based on the RMSD using the clustering tool gmx cluster of GROMACS and the GROMOS clustering algorithm.
