# Supplementary figures and images for: Exploring the Conformational Space of Bcl-2 Protein Variants: Dynamic Contributions of the Flexible Loop Domain and Transmembrane Region
Source: Molecules. 2019 Oct 29;24(21):3896. doi: 10.3390/molecules24213896 (PMC6865210; doi:10.3390/molecules24213896)

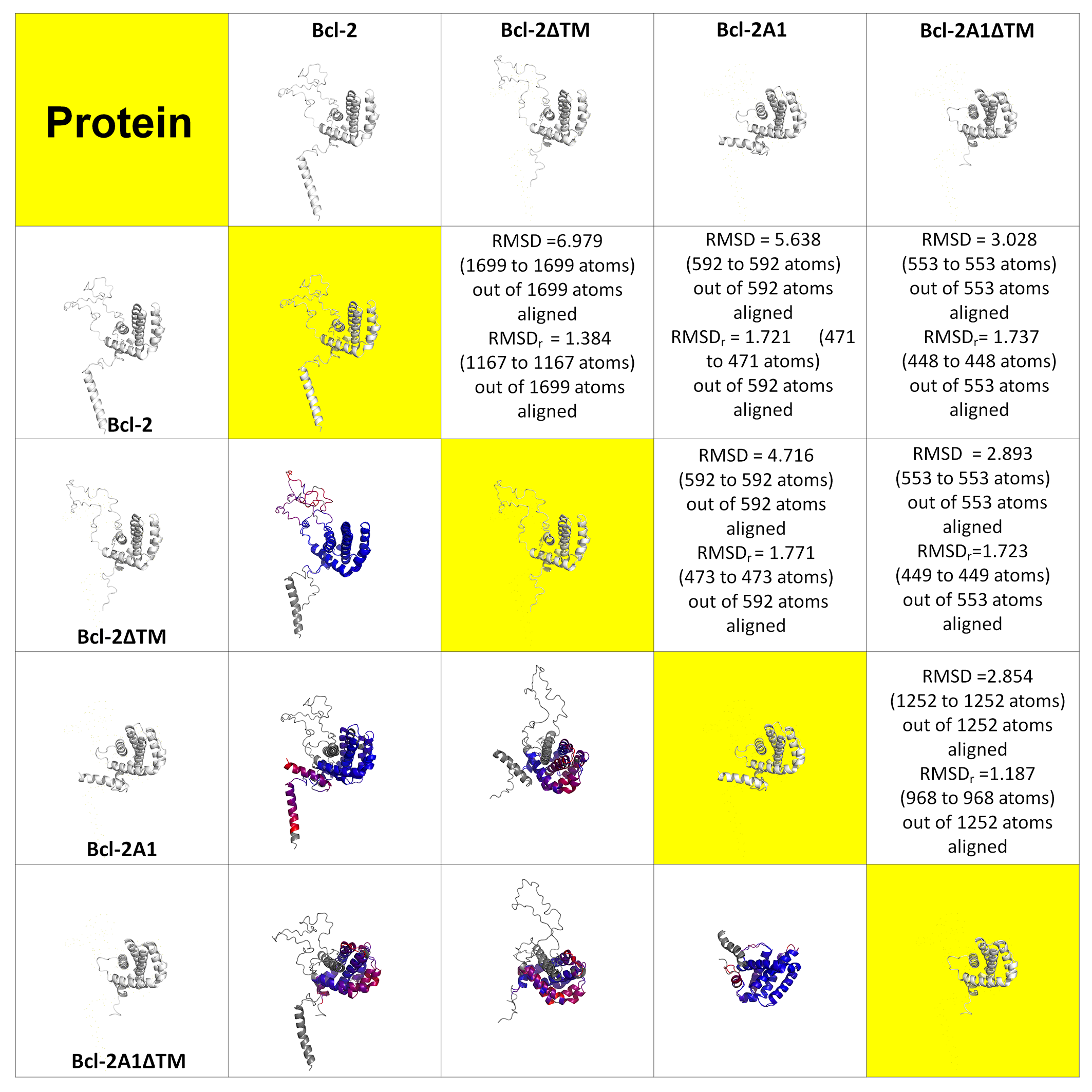

Supplement: Supplementary file 1 [file molecules-24-03896-s001.zip › Suplementaries/S1.tif]

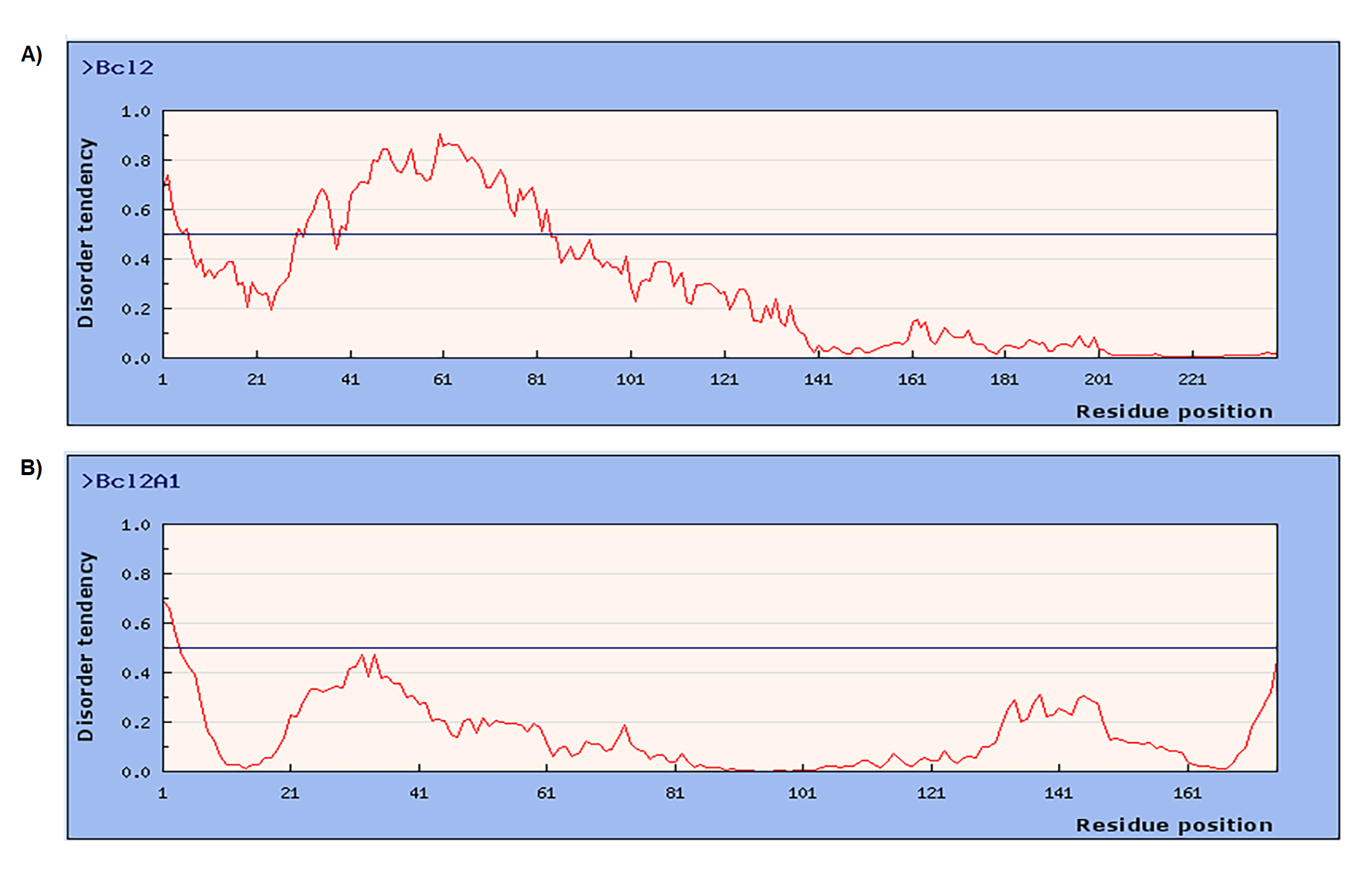

Supplement: Supplementary file 1 [file molecules-24-03896-s001.zip › Suplementaries/S10.tif]

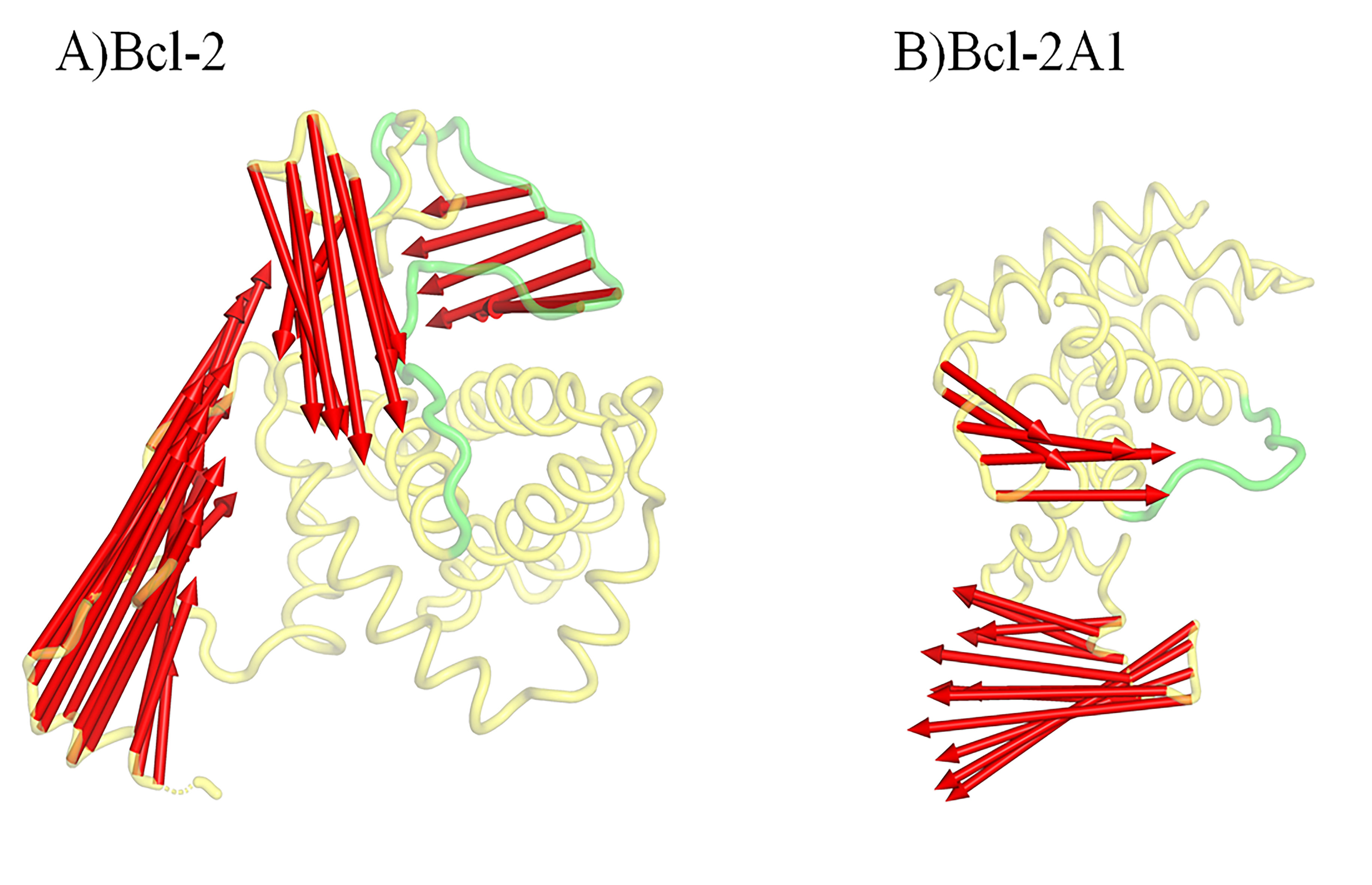

Supplement: Supplementary file 1 [file molecules-24-03896-s001.zip › Suplementaries/S11.tif]

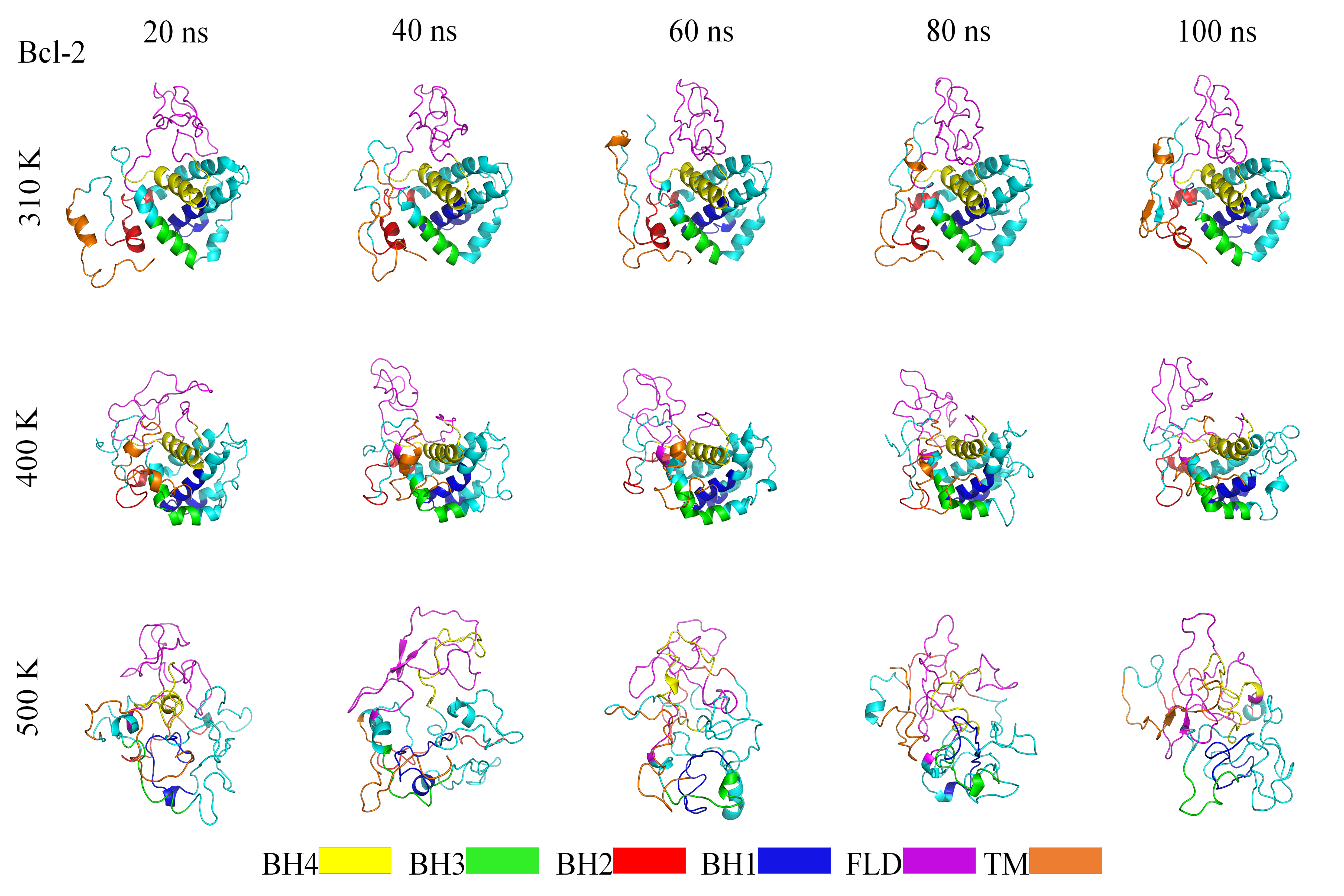

Supplement: Supplementary file 1 [file molecules-24-03896-s001.zip › Suplementaries/S2.tif]

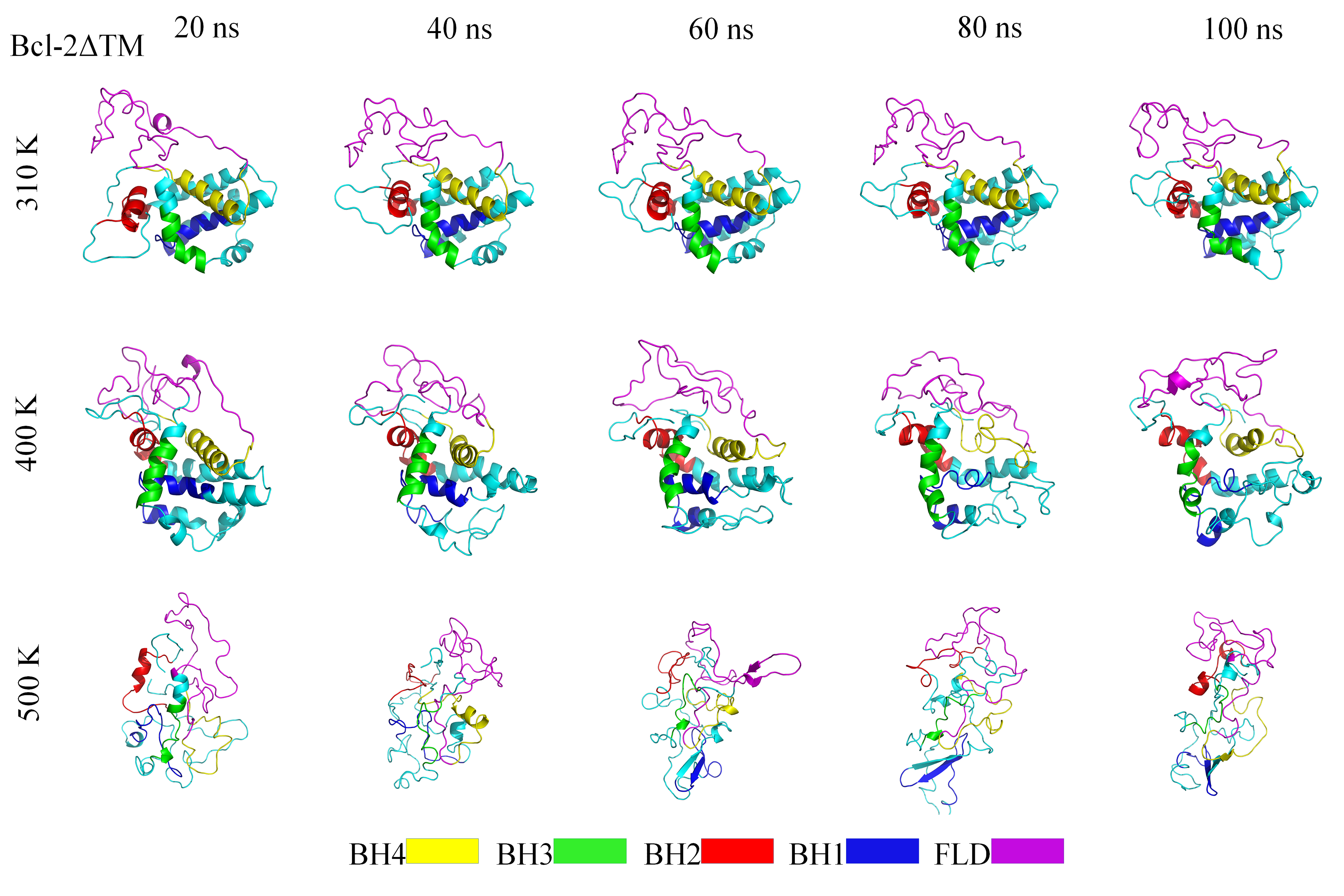

Supplement: Supplementary file 1 [file molecules-24-03896-s001.zip › Suplementaries/S3.tif]

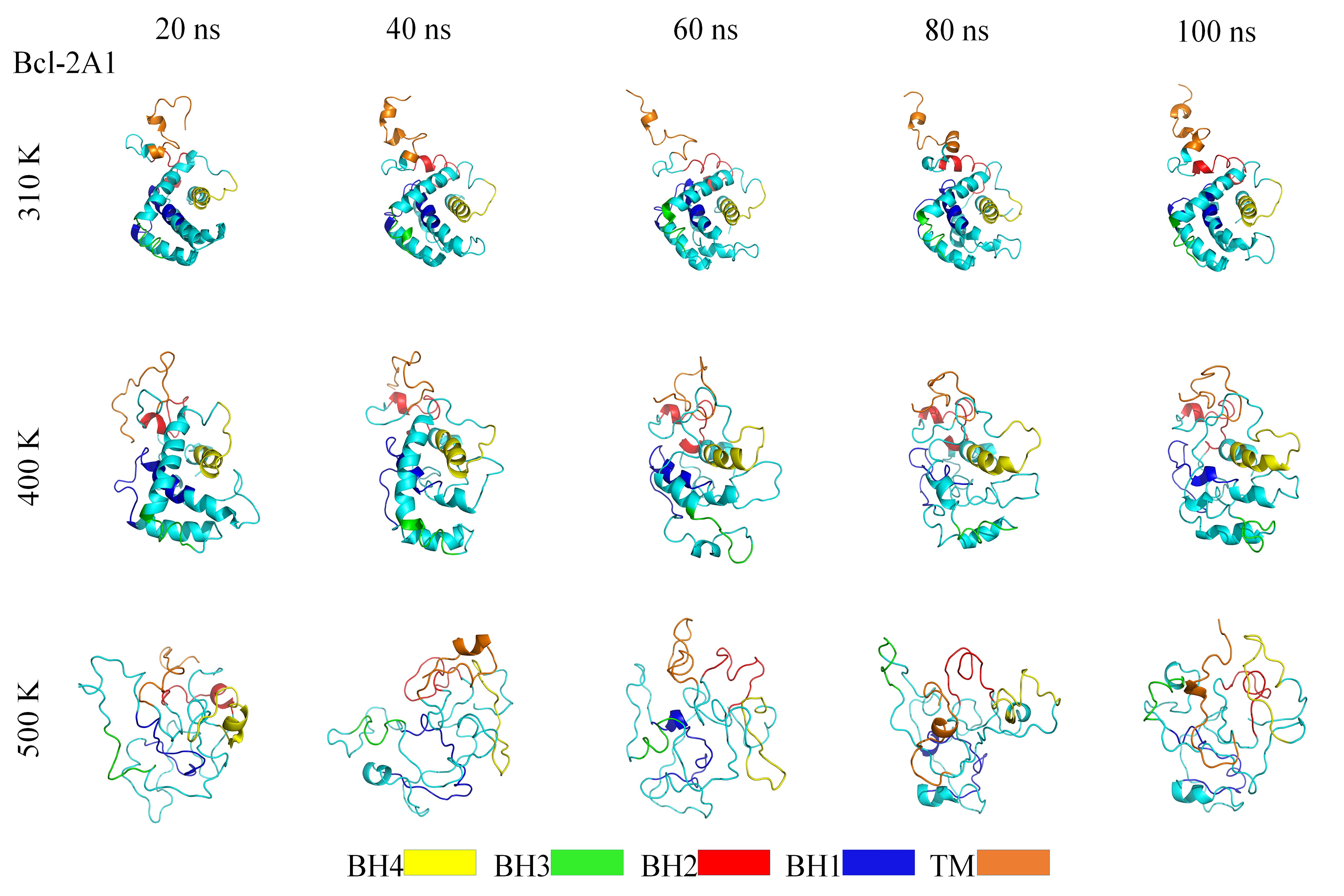

Supplement: Supplementary file 1 [file molecules-24-03896-s001.zip › Suplementaries/S4.tif]

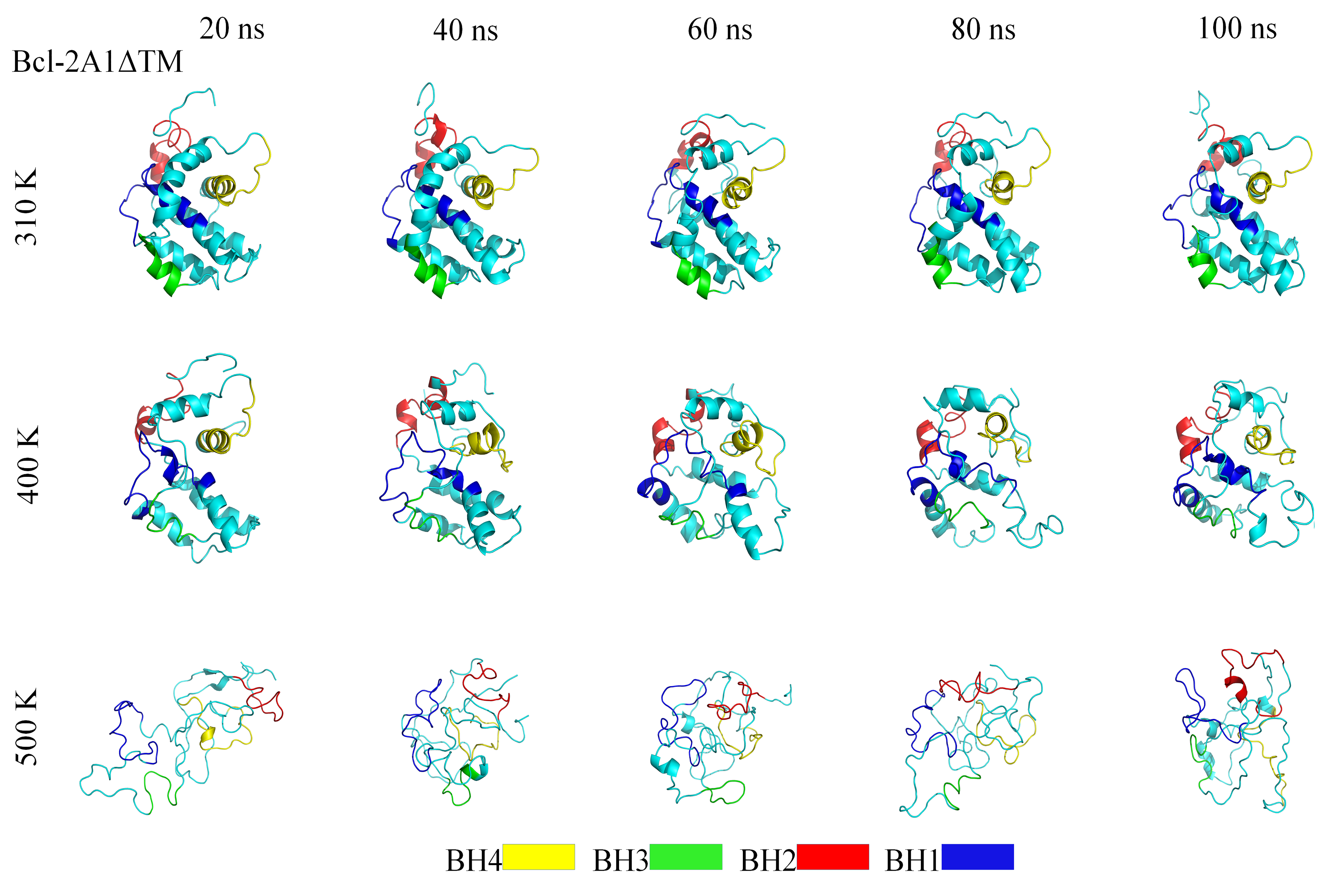

Supplement: Supplementary file 1 [file molecules-24-03896-s001.zip › Suplementaries/S5.tif]

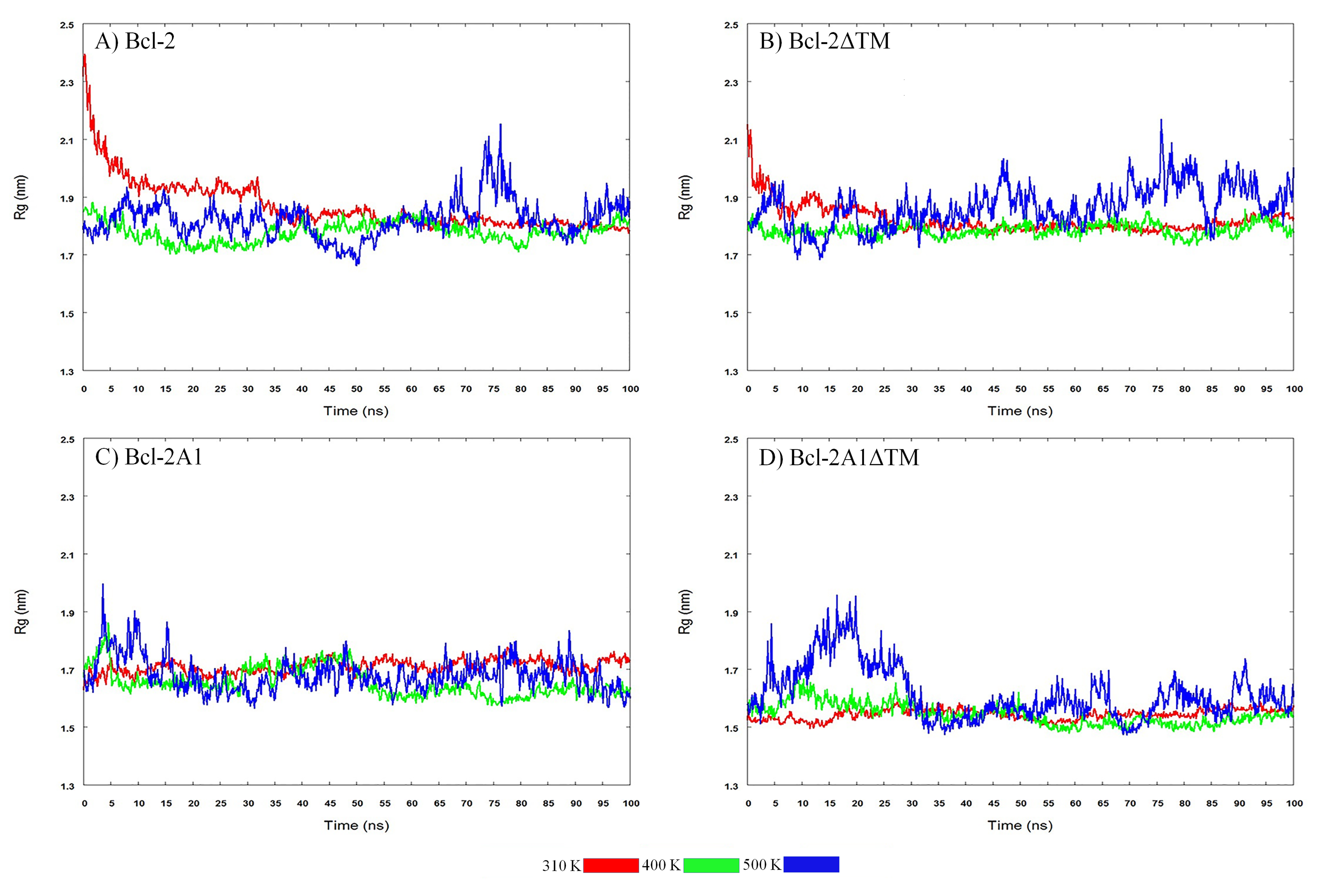

Supplement: Supplementary file 1 [file molecules-24-03896-s001.zip › Suplementaries/S6.tif]

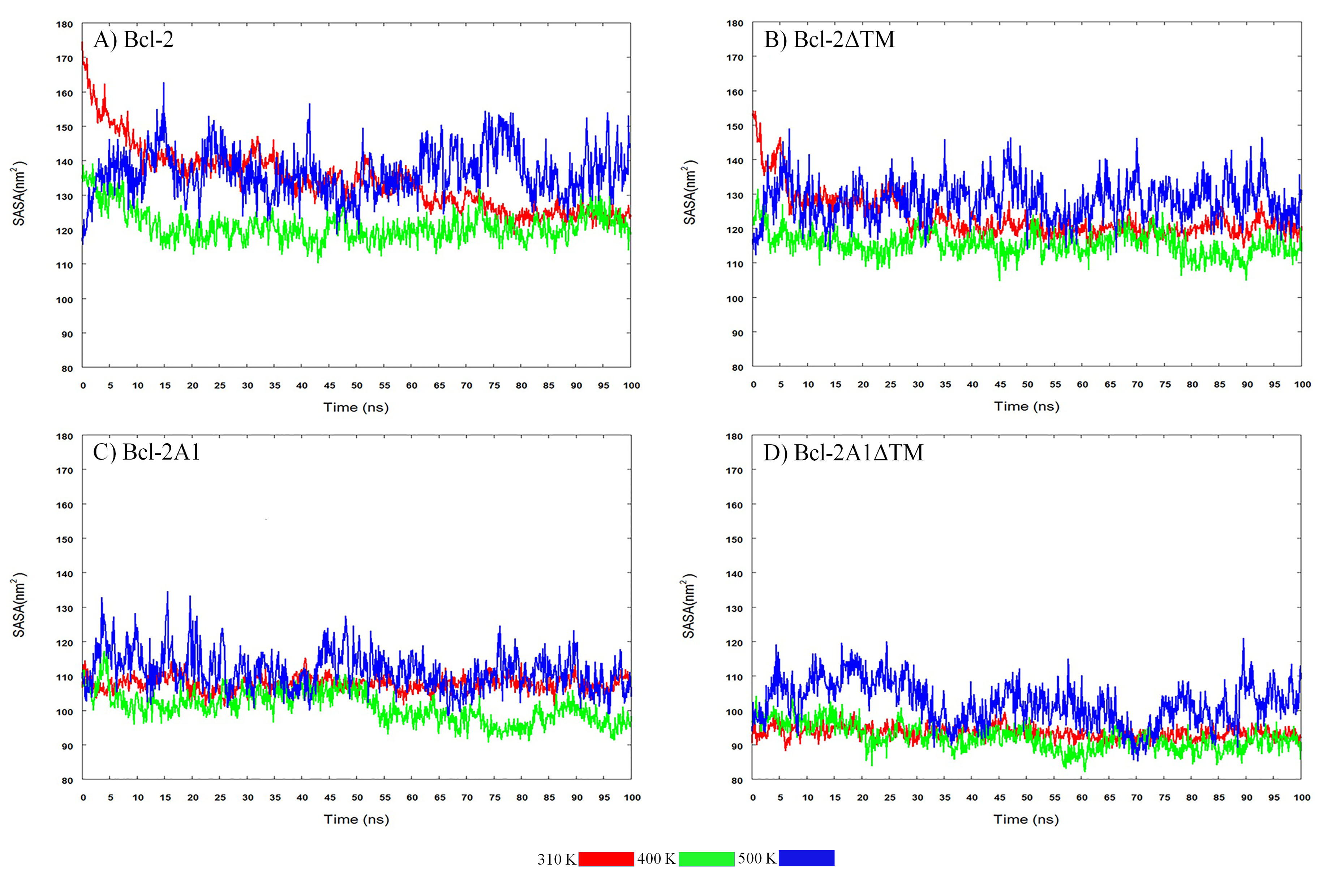

Supplement: Supplementary file 1 [file molecules-24-03896-s001.zip › Suplementaries/S7.tif]

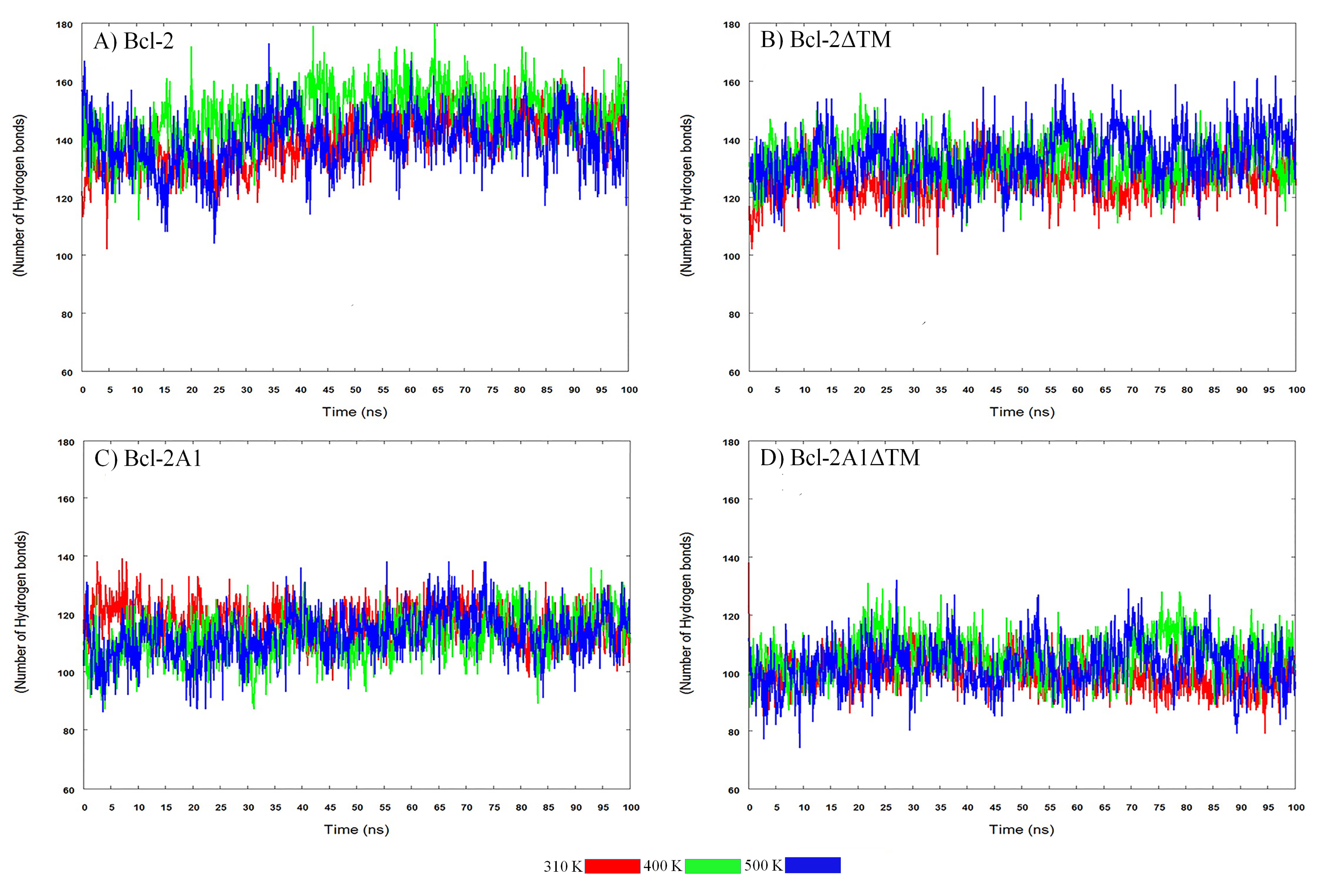

Supplement: Supplementary file 1 [file molecules-24-03896-s001.zip › Suplementaries/S8.tif]

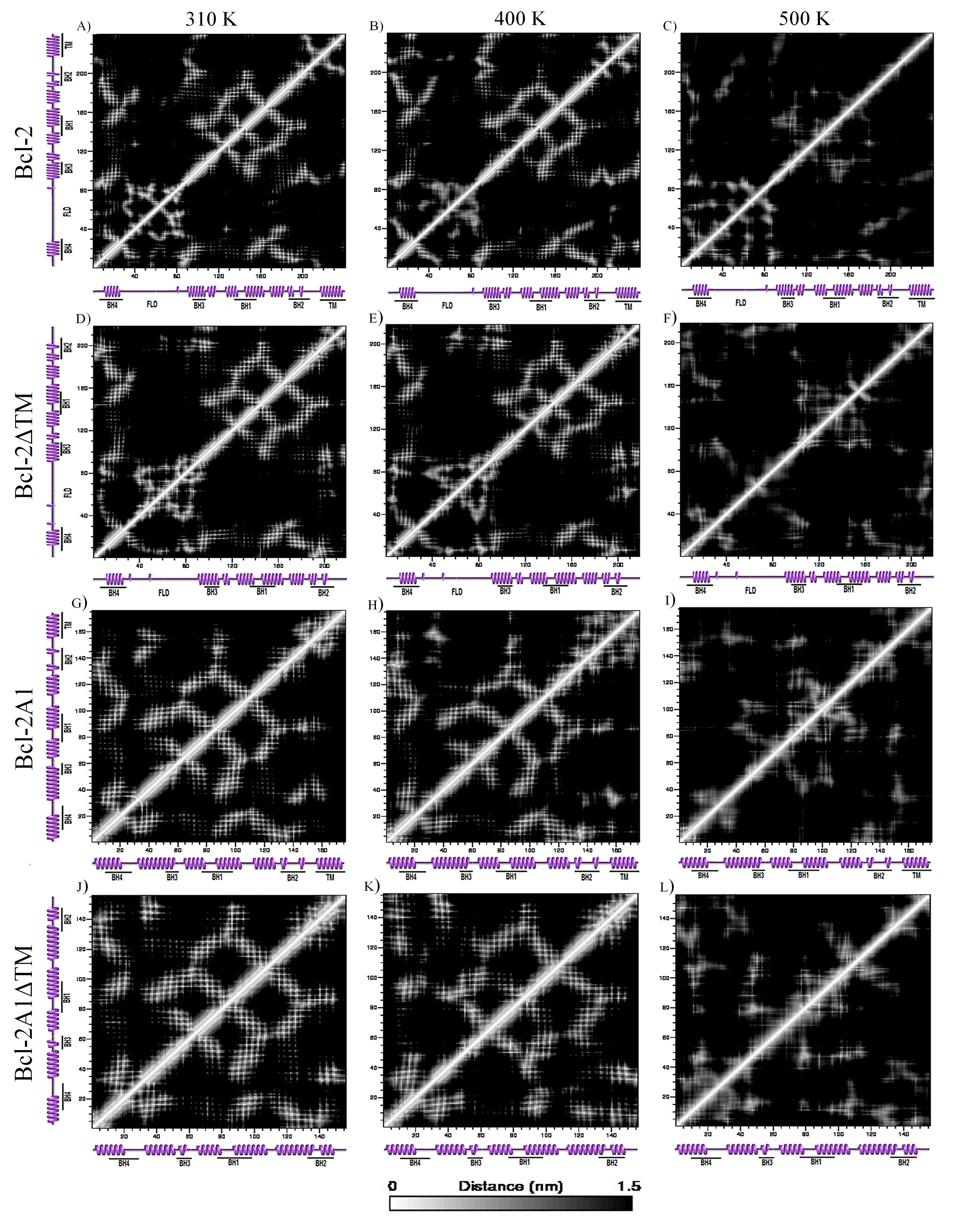

Supplement: Supplementary file 1 [file molecules-24-03896-s001.zip › Suplementaries/S9.tif]
